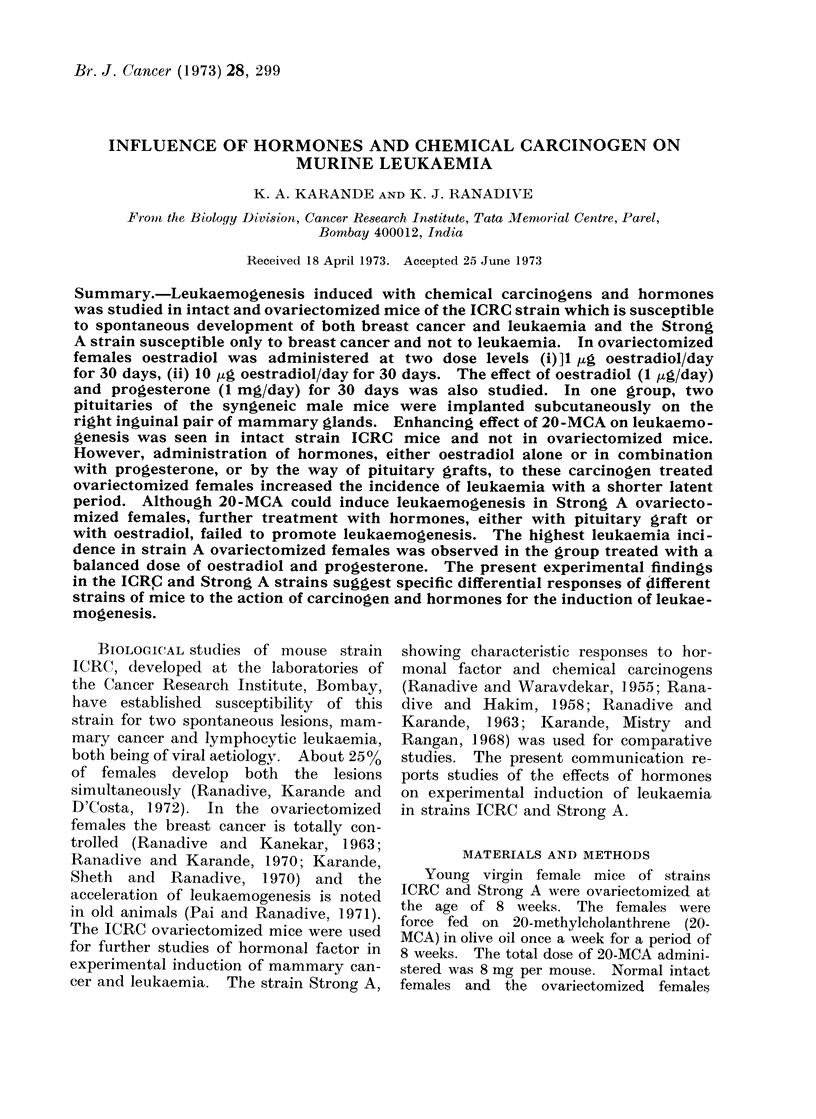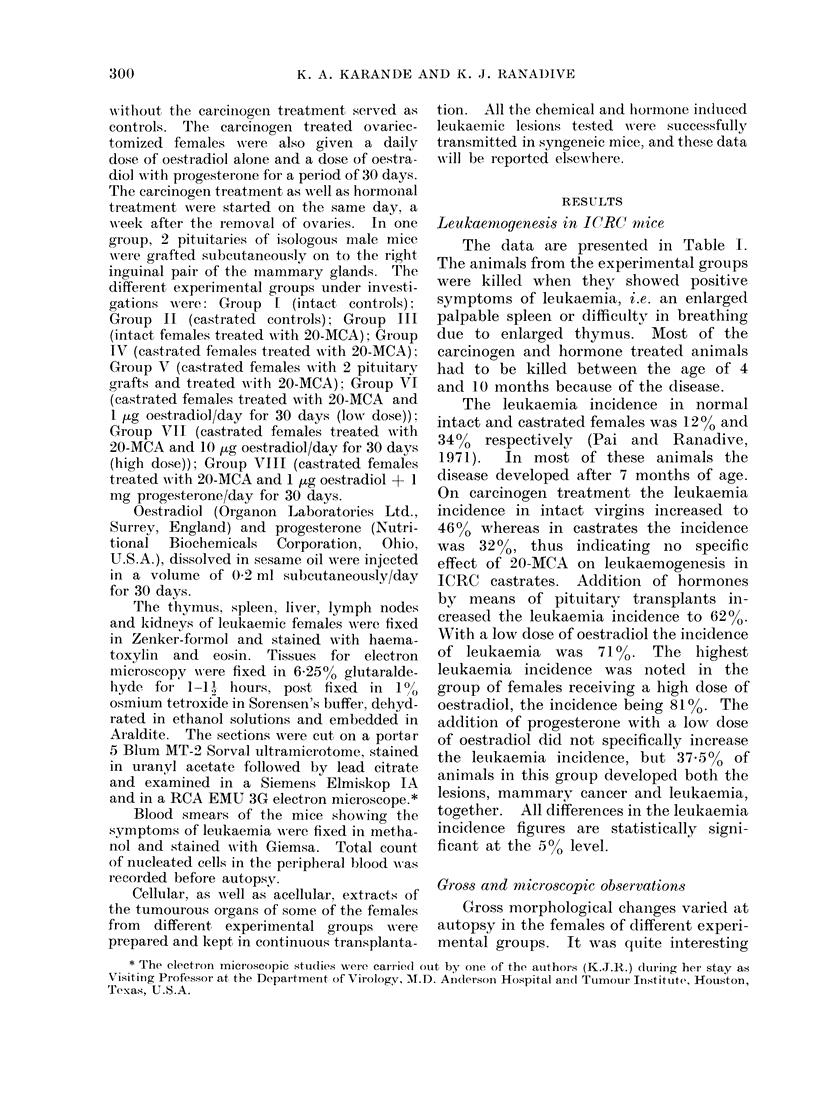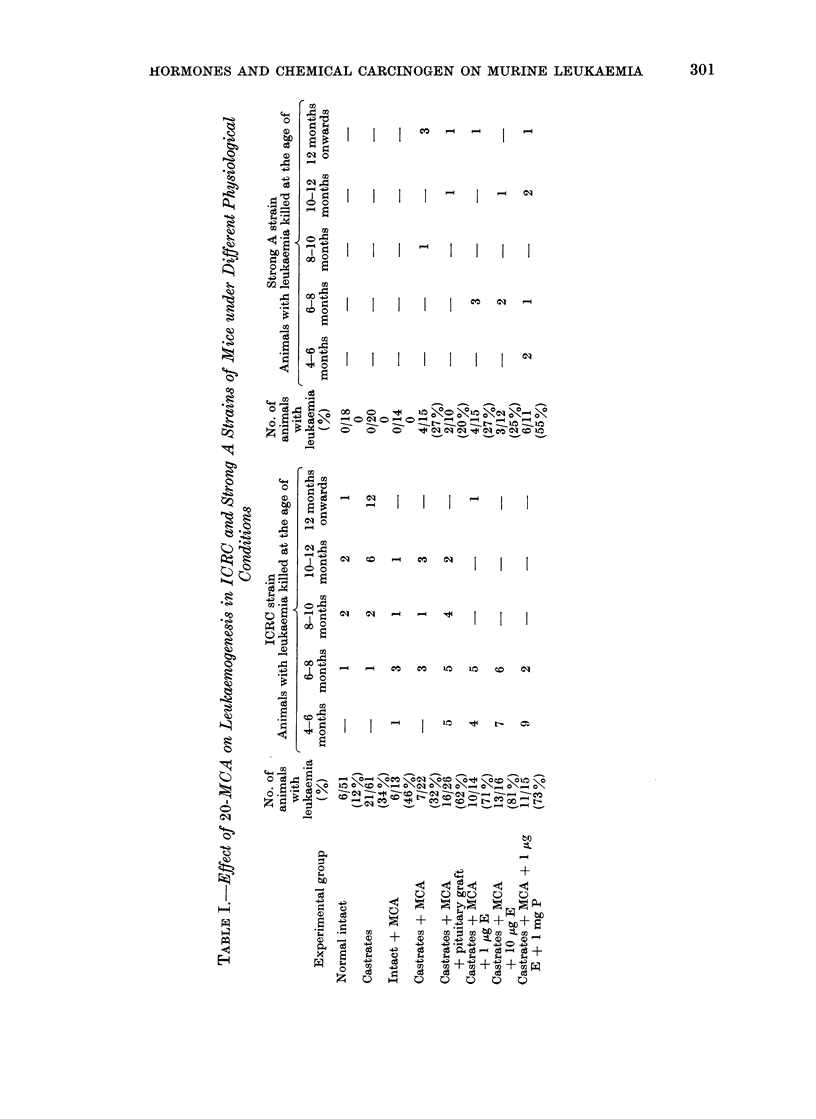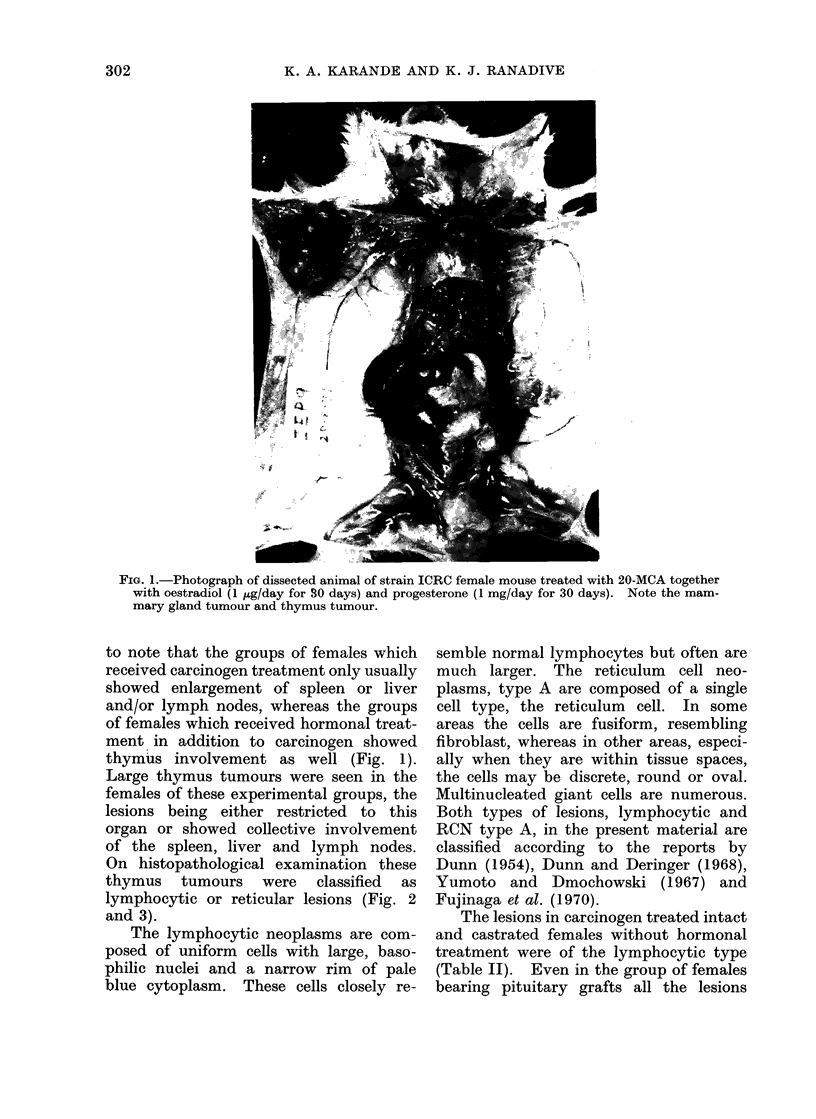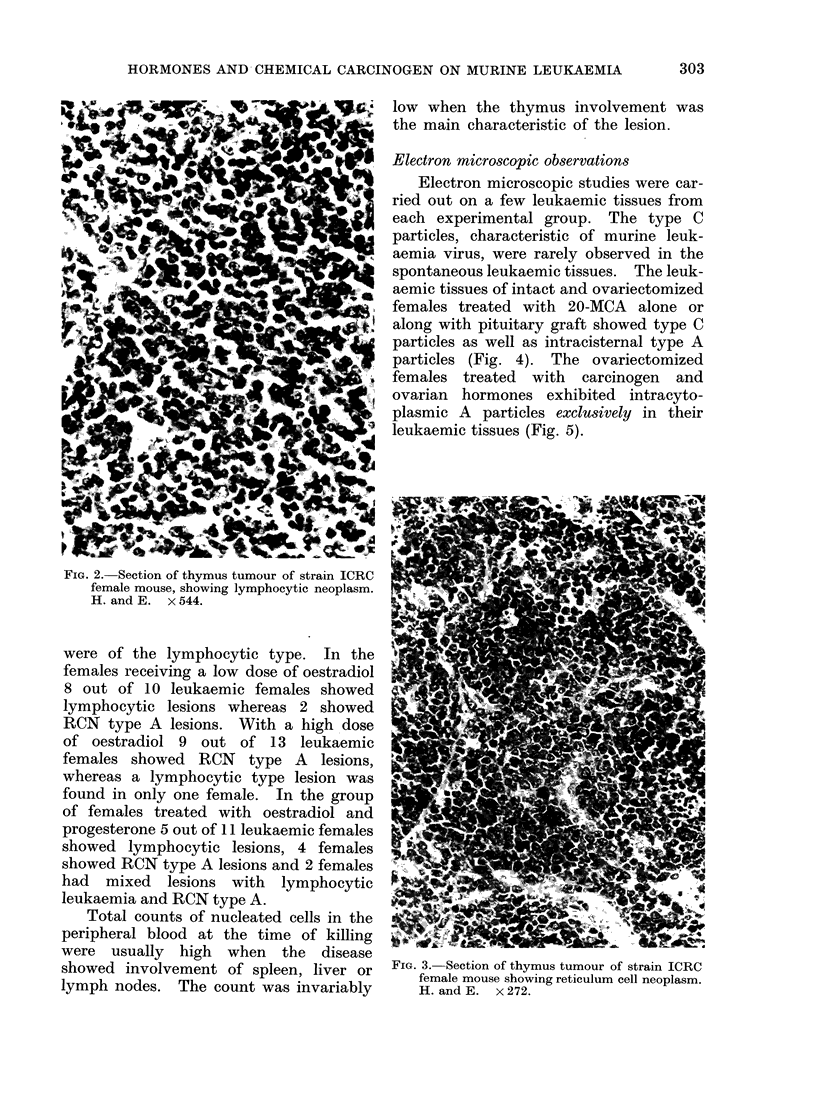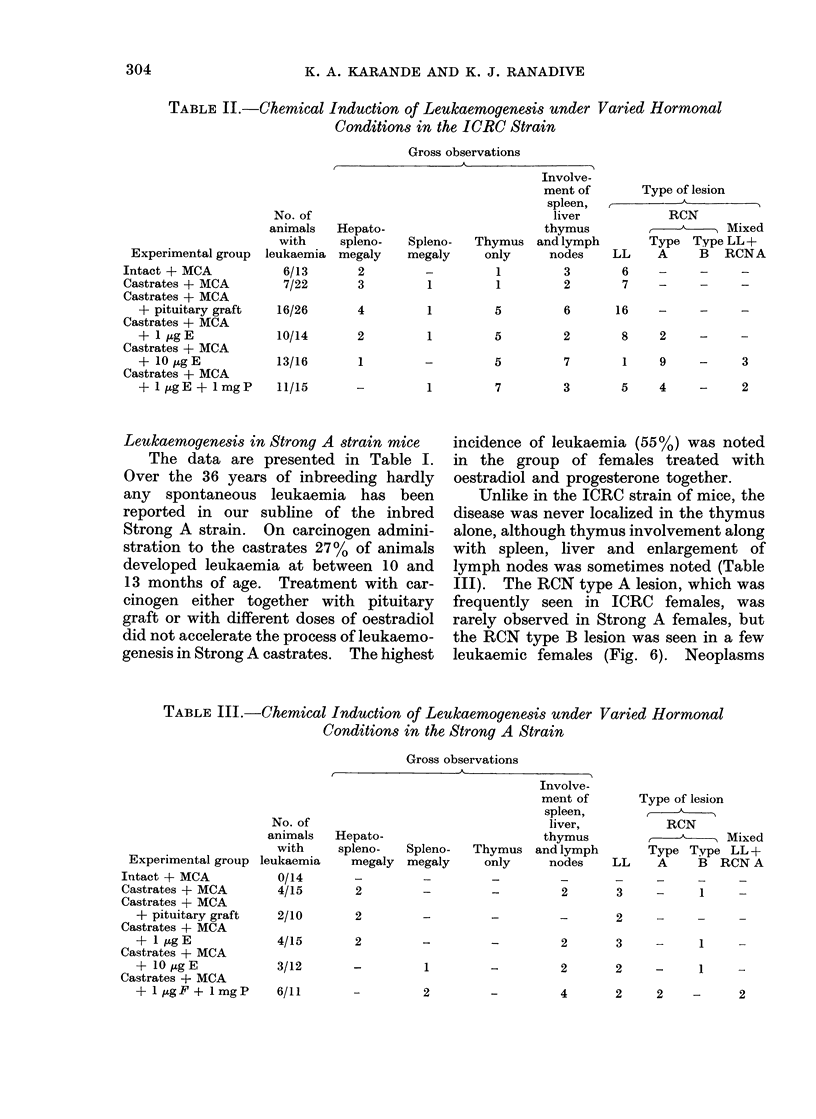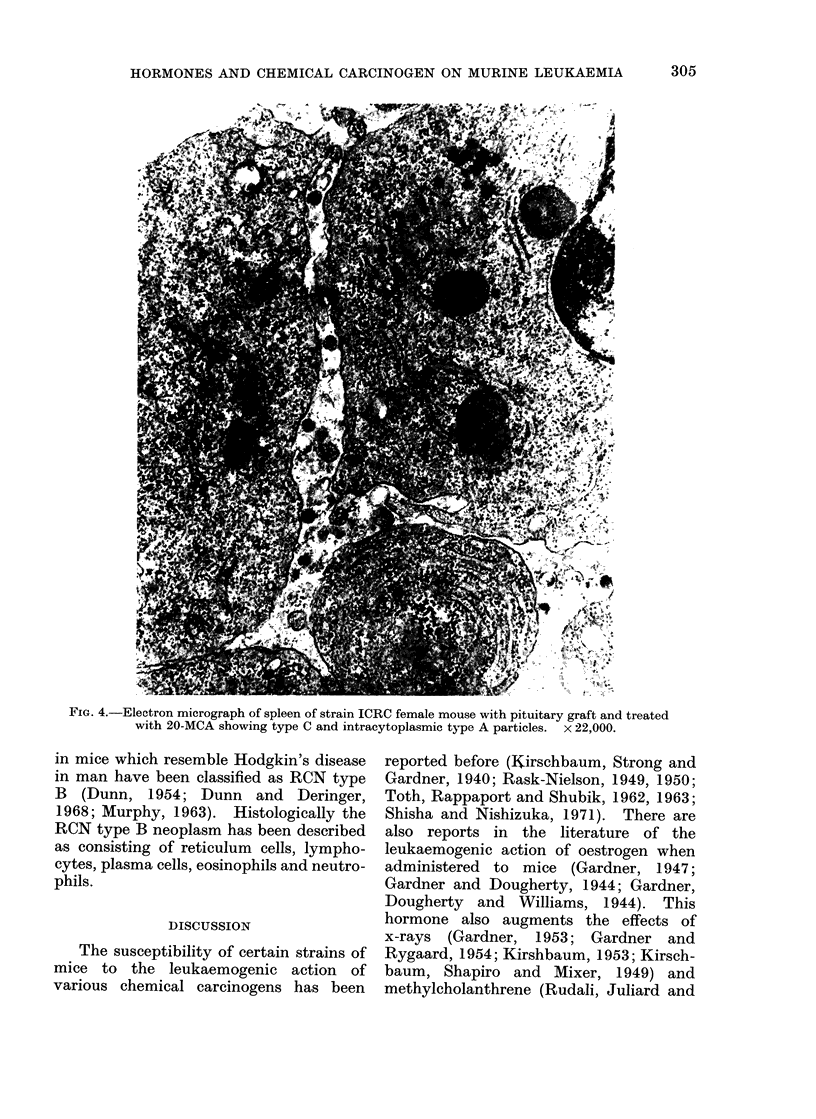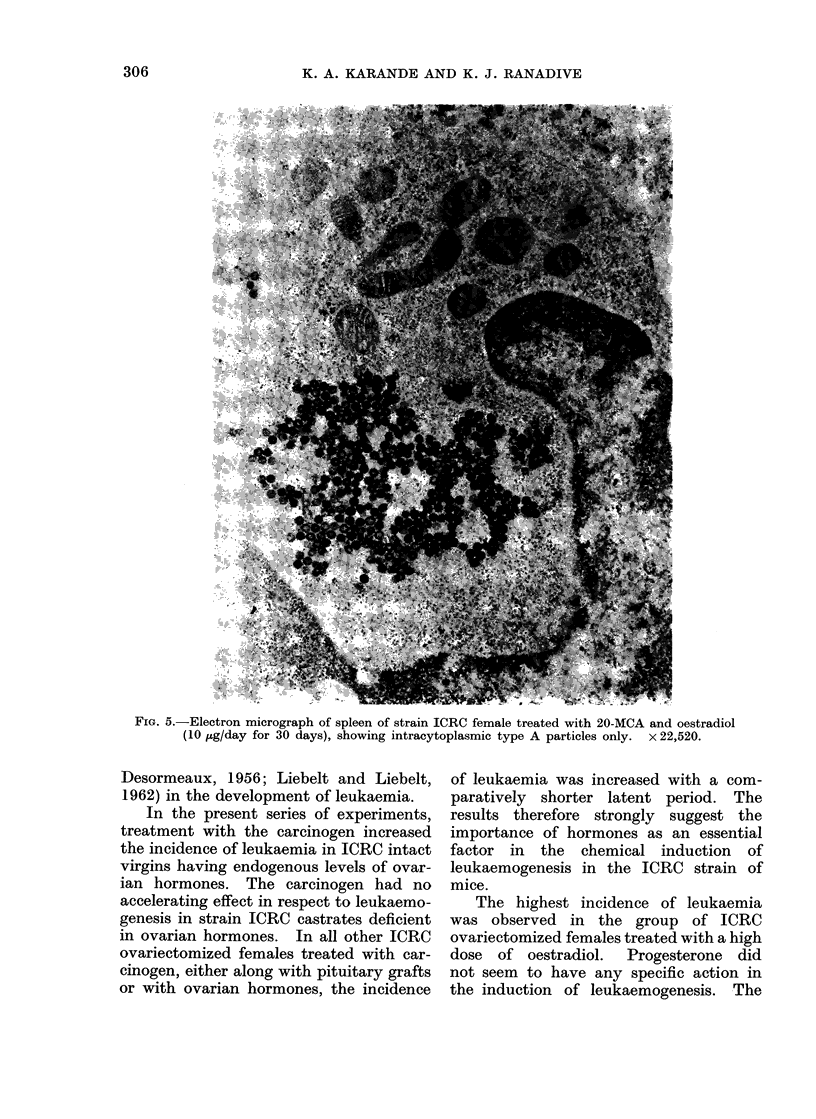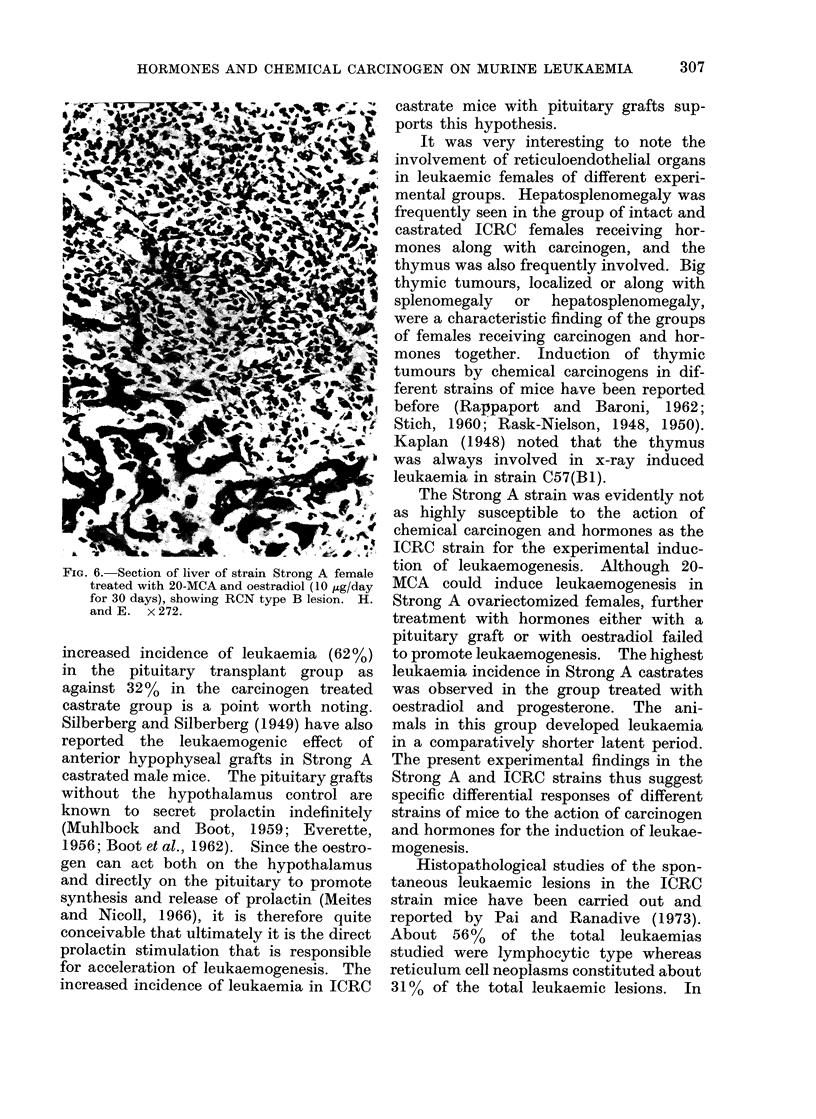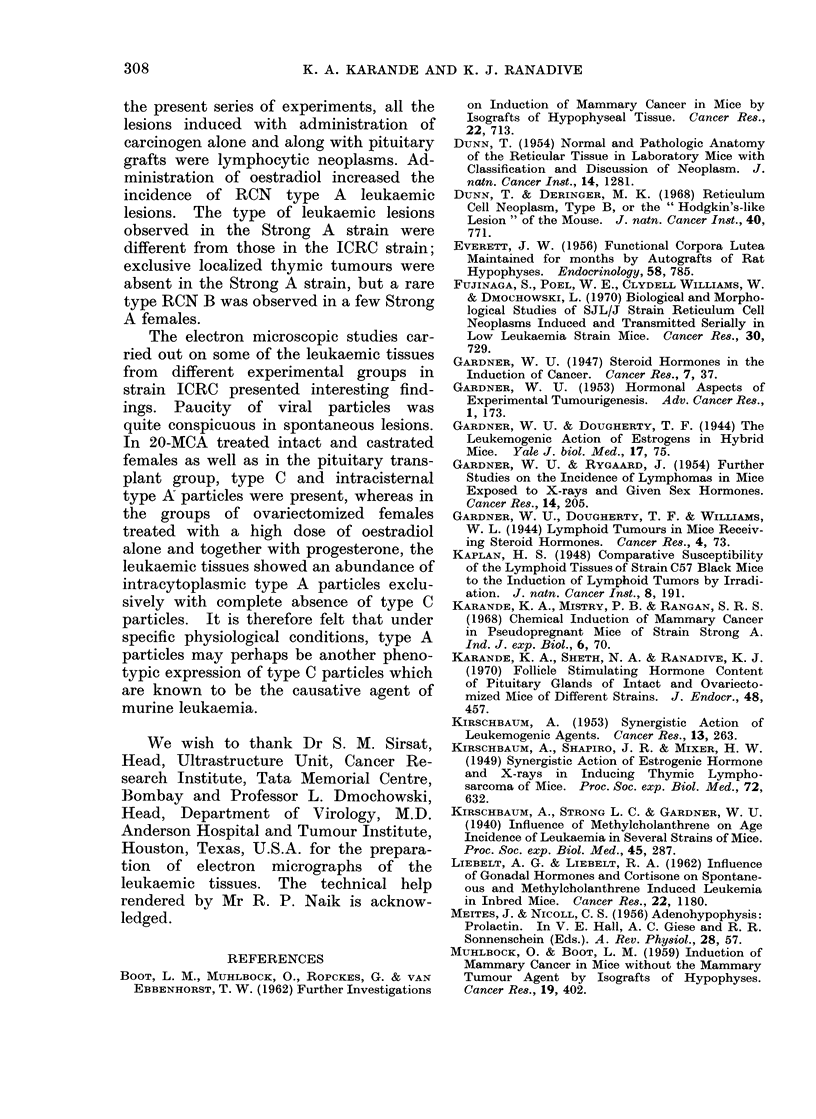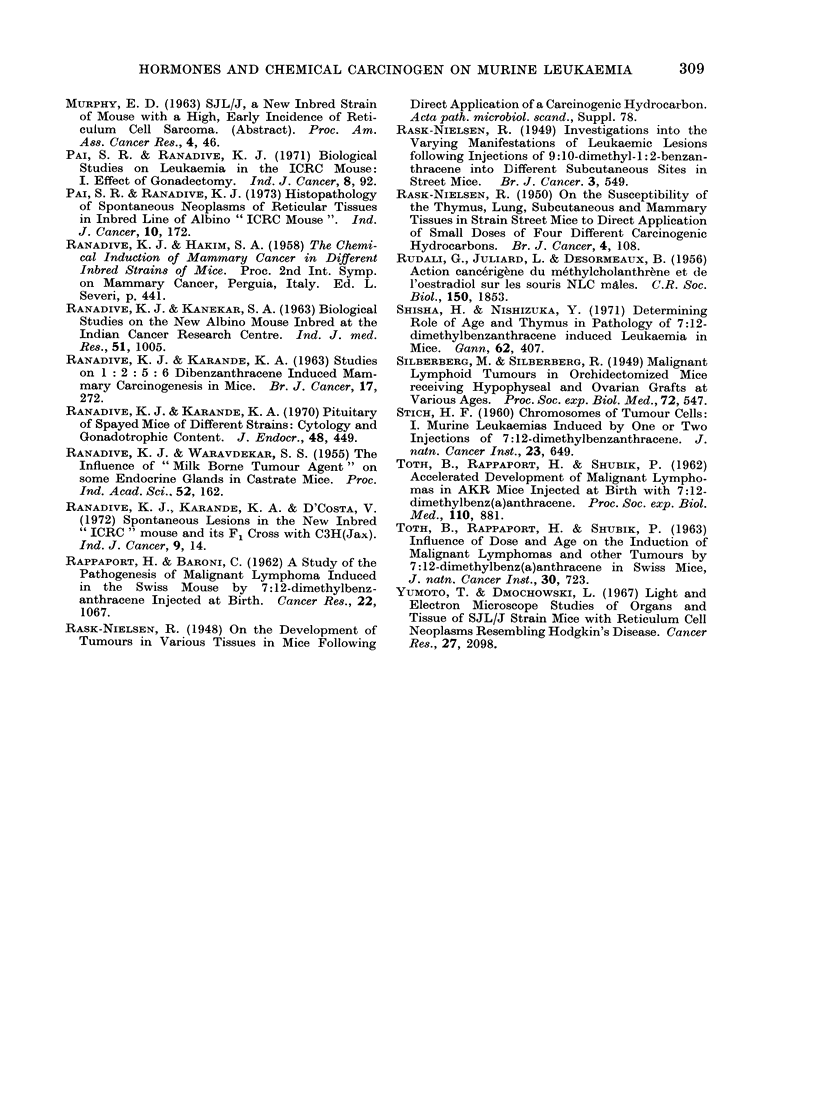# Influence of Hormones and Chemical Carcinogen on Murine Leukaemia

**DOI:** 10.1038/bjc.1973.152

**Published:** 1973-10

**Authors:** K. A. Karande, K. J. Ranadive

## Abstract

**Images:**


*Br. J. Cancer* (1973) **28**, 299

### INFLUENCE OF HORMONES AND CHEMICAL CARCINOGEN ON MURINE LEUKAEMIA

K. A. KARANDE AND K. J. RANADIVE

*From the Biology Division, Cancer Research Institute, Tata Memorial Centre, Parel, Bombay* 400012, *India*

Received 18 April 1973. Accepted 25 June 1973

**Summary.—Leukaemogenesis induced with chemical carcinogens and hormones was studied in intact and ovariectomized mice of the ICRC strain which is susceptible to spontaneous development of both breast cancer and leukaemia and the Strong A strain susceptible only to breast cancer and not to leukaemia. In ovariectomized females oestradiol was administered at two dose levels (i)]1 μg oestradiol/day for 30 days, (ii) 10 μg oestradiol/day for 30 days. The effect of oestradiol (1 μg/day) and progesterone (1 mg/day) for 30 days was also studied. In one group, two pituitaries of the syngeneic male mice were implanted subcutaneously on the right inguinal pair of mammary glands. Enhancing effect of 20-MCA on leukaemogenesis was seen in intact strain ICRC mice and not in ovariectomized mice. However, administration of hormones, either oestradiol alone or in combination with progesterone, or by the way of pituitary grafts, to these carcinogen treated ovariectomized females increased the incidence of leukaemia with a shorter latent period. Although 20-MCA could induce leukaemogenesis in Strong A ovariectomized females, further treatment with hormones, either with pituitary graft or with oestradiol, failed to promote leukaemogenesis. The highest leukaemia incidence in strain A ovariectomized females was observed in the group treated with a balanced dose of oestradiol and progesterone. The present experimental findings in the ICRC and Strong A strains suggest specific differential responses of different strains of mice to the action of carcinogen and hormones for the induction of leukaemogenesis.**

BIOLOGICAL studies of mouse strain ICRC, developed at the laboratories of the Cancer Research Institute, Bombay, have established susceptibility of this strain for two spontaneous lesions, mammary cancer and lymphocytic leukaemia, both being of viral aetiology. About 25% of females develop both the lesions simultaneously (Ranadive, Karande and D'Costa, 1972). In the ovariectomized females the breast cancer is totally controlled (Ranadive and Kanekar, 1963; Ranadive and Karande, 1970; Karande, Sheth and Ranadive, 1970) and the acceleration of leukaemogenesis is noted in old animals (Pai and Ranadive, 1971). The ICRC ovariectomized mice were used for further studies of hormonal factor in experimental induction of mammary cancer and leukaemia. The strain Strong A, showing characteristic responses to hormonal factor and chemical carcinogens (Ranadive and Waravdekar, 1955; Ranadive and Hakim, 1958; Ranadive and Karande, 1963; Karande, Mistry and Rangan, 1968) was used for comparative studies. The present communication reports studies of the effects of hormones on experimental induction of leukaemia in strains ICRC and Strong A.

### MATERIALS AND METHODS

Young virgin female mice of strains ICRC and Strong A were ovariectomized at the age of 8 weeks. The females were force fed on 20-methylcholanthrene (20-MCA) in olive oil once a week for a period of 8 weeks. The total dose of 20-MCA administered was 8 mg per mouse. Normal intact females and the ovariectomized females without the carcinogen treatment served as controls. The carcinogen treated ovariectomized females were also given a daily dose of oestradiol alone and a dose of oestradiol with progesterone for a period of 30 days. The carcinogen treatment as well as hormonal treatment were started on the same day, a week after the removal of ovaries. In one group, 2 pituitaries of isologous male mice were grafted subcutaneously on to the right inguinal pair of the mammary glands. The different experimental groups under investigations were: Group I (intact controls); Group II (castrated controls); Group III (intact females treated with 20-MCA); Group IV (castrated females treated with 20-MCA); Group V (castrated females with 2 pituitary grafts and treated with 20-MCA); Group VI (castrated females treated with 20-MCA and 1 $\mu$g oestradiol/day for 30 days (low dose)); Group VII (castrated females treated with 20-MCA and 10 $\mu$g oestradiol/day for 30 days (high dose)); Group VIII (castrated females treated with 20-MCA and 1 $\mu$g oestradiol + 1 mg progesterone/day for 30 days.

Oestradiol (Organon Laboratories Ltd., Surrey, England) and progesterone (Nutritional Biochemicals Corporation, Ohio, U.S.A.), dissolved in sesame oil were injected in a volume of 0·2 ml subcutaneously/day for 30 days.

The thymus, spleen, liver, lymph nodes and kidneys of leukaemic females were fixed in Zenker-formol and stained with haematoxylin and eosin. Tissues for electron microscopy were fixed in 6·25% glutaraldehyde for 1–1½ hours, post fixed in 1% osmium tetroxide in Sorensen's buffer, dehydrated in ethanol solutions and embedded in Araldite. The sections were cut on a portar 5 Blum MT-2 Sorval ultramicrotome, stained in uranyl acetate followed by lead citrate and examined in a Siemens Elmiskop IA and in a RCA EMU 3G electron microscope.*

Blood smears of the mice showing the symptoms of leukaemia were fixed in methanol and stained with Giemsa. Total count of nucleated cells in the peripheral blood was recorded before autopsy.

Cellular, as well as acellular, extracts of the tumourous organs of some of the females from different experimental groups were prepared and kept in continuous transplantation. All the chemical and hormone induced leukaemic lesions tested were successfully transmitted in syngeneic mice, and these data will be reported elsewhere.

### RESULTS

### *Leukaemogenesis in ICRC mice*

The data are presented in Table I. The animals from the experimental groups were killed when they showed positive symptoms of leukaemia, *i.e.* an enlarged palpable spleen or difficulty in breathing due to enlarged thymus. Most of the carcinogen and hormone treated animals had to be killed between the age of 4 and 10 months because of the disease.

The leukaemia incidence in normal intact and castrated females was 12% and 34% respectively (Pai and Ranadive, 1971). In most of these animals the disease developed after 7 months of age. On carcinogen treatment the leukaemia incidence in intact virgins increased to 46% whereas in castrates the incidence was 32%, thus indicating no specific effect of 20-MCA on leukaemogenesis in ICRC castrates. Addition of hormones by means of pituitary transplants increased the leukaemia incidence to 62%. With a low dose of oestradiol the incidence of leukaemia was 71%. The highest leukaemia incidence was noted in the group of females receiving a high dose of oestradiol, the incidence being 81%. The addition of progesterone with a low dose of oestradiol did not specifically increase the leukaemia incidence, but 37·5% of animals in this group developed both the lesions, mammary cancer and leukaemia, together. All differences in the leukaemia incidence figures are statistically significant at the 5% level.

### *Gross and microscopic observations*

Gross morphological changes varied at autopsy in the females of different experimental groups. It was quite interesting

* The electron microscopic studies were carried out by one of the authors (K.J.R.) during her stay as Visiting Professor at the Department of Virology, M.D. Anderson Hospital and Tumour Institute, Houston, Texas, U.S.A.

TABLE I.—*Effect of 20-MCA on Leukaemogenesis in ICRC and Strong A Strains of Mice under Different Physiological Conditions*

| Experimental group | ICRC strain: No. of animals with leukaemia (%) | ICRC strain: Animals with leukaemia killed at the age of 4–6 months | 6–8 months | 8–10 months | 10–12 months | 12 months onwards | Strong A strain: No. of animals with leukaemia (%) | Strong A strain: Animals with leukaemia killed at the age of 4–6 months | 6–8 months | 8–10 months | 10–12 months | 12 months onwards |
|---|---|---|---|---|---|---|---|---|---|---|---|---|
| Normal intact | 6/51 (12%) | — | 1 | 2 | 2 | 1 | 0/18 0 | — | — | — | — | — |
| Castrates | 21/61 (34%) | — | 1 | 2 | 6 | 12 | 0/20 0 | — | — | — | — | — |
| Intact + MCA | 6/13 (46%) | 1 | 3 | 1 | 1 | — | 0/14 0 | — | — | — | — | — |
| Castrates + MCA | 7/22 (32%) | — | 3 | 1 | 3 | — | 4/15 (27%) | — | — | 1 | — | 3 |
| Castrates + MCA + pituitary graft | 16/26 (62%) | 5 | 5 | 4 | 2 | — | 2/10 (20%) | — | — | — | 1 | 1 |
| Castrates + MCA + 1 μg E | 10/14 (71%) | 4 | 5 | — | — | 1 | 4/15 (27%) | — | 3 | — | — | 1 |
| Castrates + MCA + 10 μg E | 13/16 (81%) | 7 | 6 | — | — | — | 3/12 (25%) | — | 2 | — | 1 | — |
| Castrates + MCA + 1 μg E + 1 mg P | 11/15 (73%) | 9 | 2 | — | — | — | 6/11 (55%) | 2 | 1 | — | 2 | 1 |

Fig. 1.—Photograph of dissected animal of strain ICRC female mouse treated with 20-MCA together with oestradiol (1 μg/day for 30 days) and progesterone (1 mg/day for 30 days). Note the mammary gland tumour and thymus tumour.

to note that the groups of females which received carcinogen treatment only usually showed enlargement of spleen or liver and/or lymph nodes, whereas the groups of females which received hormonal treatment in addition to carcinogen showed thymus involvement as well (Fig. 1). Large thymus tumours were seen in the females of these experimental groups, the lesions being either restricted to this organ or showed collective involvement of the spleen, liver and lymph nodes. On histopathological examination these thymus tumours were classified as lymphocytic or reticular lesions (Fig. 2 and 3).

The lymphocytic neoplasms are composed of uniform cells with large, basophilic nuclei and a narrow rim of pale blue cytoplasm. These cells closely resemble normal lymphocytes but often are much larger. The reticulum cell neoplasms, type A are composed of a single cell type, the reticulum cell. In some areas the cells are fusiform, resembling fibroblast, whereas in other areas, especially when they are within tissue spaces, the cells may be discrete, round or oval. Multinucleated giant cells are numerous. Both types of lesions, lymphocytic and RCN type A, in the present material are classified according to the reports by Dunn (1954), Dunn and Deringer (1968), Yumoto and Dmochowski (1967) and Fujinaga *et al.* (1970).

The lesions in carcinogen treated intact and castrated females without hormonal treatment were of the lymphocytic type (Table II). Even in the group of females bearing pituitary grafts all the lesions

FIG. 2.—Section of thymus tumour of strain ICRC female mouse, showing lymphocytic neoplasm. H. and E. ×544.

were of the lymphocytic type. In the females receiving a low dose of oestradiol 8 out of 10 leukaemic females showed lymphocytic lesions whereas 2 showed RCN type A lesions. With a high dose of oestradiol 9 out of 13 leukaemic females showed RCN type A lesions, whereas a lymphocytic type lesion was found in only one female. In the group of females treated with oestradiol and progesterone 5 out of 11 leukaemic females showed lymphocytic lesions, 4 females showed RCN type A lesions and 2 females had mixed lesions with lymphocytic leukaemia and RCN type A.

Total counts of nucleated cells in the peripheral blood at the time of killing were usually high when the disease showed involvement of spleen, liver or lymph nodes. The count was invariably low when the thymus involvement was the main characteristic of the lesion.

*Electron microscopic observations*

Electron microscopic studies were carried out on a few leukaemic tissues from each experimental group. The type C particles, characteristic of murine leukaemia virus, were rarely observed in the spontaneous leukaemic tissues. The leukaemic tissues of intact and ovariectomized females treated with 20-MCA alone or along with pituitary graft showed type C particles as well as intracisternal type A particles (Fig. 4). The ovariectomized females treated with carcinogen and ovarian hormones exhibited intracytoplasmic A particles *exclusively* in their leukaemic tissues (Fig. 5).

FIG. 3.—Section of thymus tumour of strain ICRC female mouse showing reticulum cell neoplasm. H. and E. ×272.

TABLE II.—*Chemical Induction of Leukaemogenesis under Varied Hormonal Conditions in the ICRC Strain*

| | | Gross observations | | | | Type of lesion | | | |
|---|---|---|---|---|---|---|---|---|---|
| | | | | | | | RCN | | |
| Experimental group | No. of animals with leukaemia | Hepato-spleno-megaly | Spleno-megaly | Thymus only | Involve-ment of spleen, liver thymus and lymph nodes | LL | Type A | Type B | Mixed LL + RCN A |
| Intact + MCA | 6/13 | 2 | – | 1 | 3 | 6 | – | – | – |
| Castrates + MCA | 7/22 | 3 | 1 | 1 | 2 | 7 | – | – | – |
| Castrates + MCA + pituitary graft | 16/26 | 4 | 1 | 5 | 6 | 16 | – | – | – |
| Castrates + MCA + 1 $\mu$g E | 10/14 | 2 | 1 | 5 | 2 | 8 | 2 | – | – |
| Castrates + MCA + 10 $\mu$g E | 13/16 | 1 | – | 5 | 7 | 1 | 9 | – | 3 |
| Castrates + MCA + 1 $\mu$g E + 1 mg P | 11/15 | – | 1 | 7 | 3 | 5 | 4 | – | 2 |

*Leukaemogenesis in Strong A strain mice*

The data are presented in Table I. Over the 36 years of inbreeding hardly any spontaneous leukaemia has been reported in our subline of the inbred Strong A strain. On carcinogen administration to the castrates 27% of animals developed leukaemia at between 10 and 13 months of age. Treatment with carcinogen either together with pituitary graft or with different doses of oestradiol did not accelerate the process of leukaemogenesis in Strong A castrates. The highest incidence of leukaemia (55%) was noted in the group of females treated with oestradiol and progesterone together.

Unlike in the ICRC strain of mice, the disease was never localized in the thymus alone, although thymus involvement along with spleen, liver and enlargement of lymph nodes was sometimes noted (Table III). The RCN type A lesion, which was frequently seen in ICRC females, was rarely observed in Strong A females, but the RCN type B lesion was seen in a few leukaemic females (Fig. 6). Neoplasms

TABLE III.—*Chemical Induction of Leukaemogenesis under Varied Hormonal Conditions in the Strong A Strain*

| | | Gross observations | | | | Type of lesion | | | |
|---|---|---|---|---|---|---|---|---|---|
| | | | | | | | RCN | | |
| Experimental group | No. of animals with leukaemia | Hepato-spleno-megaly | Spleno-megaly | Thymus only | Involve-ment of spleen, liver, thymus and lymph nodes | LL | Type A | Type B | Mixed LL + RCN A |
| Intact + MCA | 0/14 | – | – | – | – | – | – | – | – |
| Castrates + MCA | 4/15 | 2 | – | – | 2 | 3 | – | 1 | – |
| Castrates + MCA + pituitary graft | 2/10 | 2 | – | – | – | 2 | – | – | – |
| Castrates + MCA + 1 $\mu$g E | 4/15 | 2 | – | – | 2 | 3 | – | 1 | – |
| Castrates + MCA + 10 $\mu$g E | 3/12 | – | 1 | – | 2 | 2 | – | 1 | – |
| Castrates + MCA + 1 $\mu$g *F* + 1 mg P | 6/11 | – | 2 | – | 4 | 2 | 2 | – | 2 |

FIG. 4.—Electron micrograph of spleen of strain ICRC female mouse with pituitary graft and treated with 20-MCA showing type C and intracytoplasmic type A particles. ×22,000.

in mice which resemble Hodgkin's disease in man have been classified as RCN type B (Dunn, 1954; Dunn and Deringer, 1968; Murphy, 1963). Histologically the RCN type B neoplasm has been described as consisting of reticulum cells, lymphocytes, plasma cells, eosinophils and neutrophils.

### DISCUSSION

The susceptibility of certain strains of mice to the leukaemogenic action of various chemical carcinogens has been reported before (Kirschbaum, Strong and Gardner, 1940; Rask-Nielson, 1949, 1950; Toth, Rappaport and Shubik, 1962, 1963; Shisha and Nishizuka, 1971). There are also reports in the literature of the leukaemogenic action of oestrogen when administered to mice (Gardner, 1947; Gardner and Dougherty, 1944; Gardner, Dougherty and Williams, 1944). This hormone also augments the effects of x-rays (Gardner, 1953; Gardner and Rygaard, 1954; Kirshbaum, 1953; Kirschbaum, Shapiro and Mixer, 1949) and methylcholanthrene (Rudali, Juliard and

FIG. 5.—Electron micrograph of spleen of strain ICRC female treated with 20-MCA and oestradiol (10 $\mu$g/day for 30 days), showing intracytoplasmic type A particles only. $\times$ 22,520.

Desormeaux, 1956; Liebelt and Liebelt, 1962) in the development of leukaemia.

In the present series of experiments, treatment with the carcinogen increased the incidence of leukaemia in ICRC intact virgins having endogenous levels of ovarian hormones. The carcinogen had no accelerating effect in respect to leukaemogenesis in strain ICRC castrates deficient in ovarian hormones. In all other ICRC ovariectomized females treated with carcinogen, either along with pituitary grafts or with ovarian hormones, the incidence of leukaemia was increased with a comparatively shorter latent period. The results therefore strongly suggest the importance of hormones as an essential factor in the chemical induction of leukaemogenesis in the ICRC strain of mice.

The highest incidence of leukaemia was observed in the group of ICRC ovariectomized females treated with a high dose of oestradiol. Progesterone did not seem to have any specific action in the induction of leukaemogenesis. The

FIG. 6.—Section of liver of strain Strong A female treated with 20-MCA and oestradiol (10 μg/day for 30 days), showing RCN type B lesion. H. and E. ×272.

increased incidence of leukaemia (62%) in the pituitary transplant group as against 32% in the carcinogen treated castrate group is a point worth noting. Silberberg and Silberberg (1949) have also reported the leukaemogenic effect of anterior hypophyseal grafts in Strong A castrated male mice. The pituitary grafts without the hypothalamus control are known to secret prolactin indefinitely (Muhlbock and Boot, 1959; Everette, 1956; Boot *et al.*, 1962). Since the oestrogen can act both on the hypothalamus and directly on the pituitary to promote synthesis and release of prolactin (Meites and Nicoll, 1966), it is therefore quite conceivable that ultimately it is the direct prolactin stimulation that is responsible for acceleration of leukaemogenesis. The increased incidence of leukaemia in ICRC castrate mice with pituitary grafts supports this hypothesis.

It was very interesting to note the involvement of reticuloendothelial organs in leukaemic females of different experimental groups. Hepatosplenomegaly was frequently seen in the group of intact and castrated ICRC females receiving hormones along with carcinogen, and the thymus was also frequently involved. Big thymic tumours, localized or along with splenomegaly or hepatosplenomegaly, were a characteristic finding of the groups of females receiving carcinogen and hormones together. Induction of thymic tumours by chemical carcinogens in different strains of mice have been reported before (Rappaport and Baroni, 1962; Stich, 1960; Rask-Nielson, 1948, 1950). Kaplan (1948) noted that the thymus was always involved in x-ray induced leukaemia in strain C57(Bl).

The Strong A strain was evidently not as highly susceptible to the action of chemical carcinogen and hormones as the ICRC strain for the experimental induction of leukaemogenesis. Although 20-MCA could induce leukaemogenesis in Strong A ovariectomized females, further treatment with hormones either with a pituitary graft or with oestradiol failed to promote leukaemogenesis. The highest leukaemia incidence in Strong A castrates was observed in the group treated with oestradiol and progesterone. The animals in this group developed leukaemia in a comparatively shorter latent period. The present experimental findings in the Strong A and ICRC strains thus suggest specific differential responses of different strains of mice to the action of carcinogen and hormones for the induction of leukaemogenesis.

Histopathological studies of the spontaneous leukaemic lesions in the ICRC strain mice have been carried out and reported by Pai and Ranadive (1973). About 56% of the total leukaemias studied were lymphocytic type whereas reticulum cell neoplasms constituted about 31% of the total leukaemic lesions. In the present series of experiments, all the lesions induced with administration of carcinogen alone and along with pituitary grafts were lymphocytic neoplasms. Administration of oestradiol increased the incidence of RCN type A leukaemic lesions. The type of leukaemic lesions observed in the Strong A strain were different from those in the ICRC strain; exclusive localized thymic tumours were absent in the Strong A strain, but a rare type RCN B was observed in a few Strong A females.

The electron microscopic studies carried out on some of the leukaemic tissues from different experimental groups in strain ICRC presented interesting findings. Paucity of viral particles was quite conspicuous in spontaneous lesions. In 20-MCA treated intact and castrated females as well as in the pituitary transplant group, type C and intracisternal type A particles were present, whereas in the groups of ovariectomized females treated with a high dose of oestradiol alone and together with progesterone, the leukaemic tissues showed an abundance of intracytoplasmic type A particles exclusively with complete absence of type C particles. It is therefore felt that under specific physiological conditions, type A particles may perhaps be another phenotypic expression of type C particles which are known to be the causative agent of murine leukaemia.

We wish to thank Dr S. M. Sirsat, Head, Ultrastructure Unit, Cancer Research Institute, Tata Memorial Centre, Bombay and Professor L. Dmochowski, Head, Department of Virology, M.D. Anderson Hospital and Tumour Institute, Houston, Texas, U.S.A. for the preparation of electron micrographs of the leukaemic tissues. The technical help rendered by Mr R. P. Naik is acknowledged.

REFERENCES

Boot, L. M., Muhlbock, O., Ropckes, G. & van Ebbenhorst, T. W. (1962) Further Investigations on Induction of Mammary Cancer in Mice by Isografts of Hypophyseal Tissue. *Cancer Res.*, **22**, 713.

Dunn, T. (1954) Normal and Pathologic Anatomy of the Reticular Tissue in Laboratory Mice with Classification and Discussion of Neoplasm. *J. natn. Cancer Inst.*, **14**, 1281.

Dunn, T. & Deringer, M. K. (1968) Reticulum Cell Neoplasm, Type B, or the " Hodgkin's-like Lesion " of the Mouse. *J. natn. Cancer Inst.*, **40**, 771.

Everett, J. W. (1956) Functional Corpora Lutea Maintained for months by Autografts of Rat Hypophyses. *Endocrinology*, **58**, 785.

Fujinaga, S., Poel, W. E., Clydell Williams, W. & Dmochowski, L. (1970) Biological and Morphological Studies of SJL/J Strain Reticulum Cell Neoplasms Induced and Transmitted Serially in Low Leukaemia Strain Mice. *Cancer Res.*, **30**, 729.

Gardner, W. U. (1947) Steroid Hormones in the Induction of Cancer. *Cancer Res.*, **7**, 37.

Gardner, W. U. (1953) Hormonal Aspects of Experimental Tumourigenesis. *Adv. Cancer Res.*, **1**, 173.

Gardner, W. U. & Dougherty, T. F. (1944) The Leukemogenic Action of Estrogens in Hybrid Mice. *Yale J. biol. Med.*, **17**, 75.

Gardner, W. U. & Rygaard, J. (1954) Further Studies on the Incidence of Lymphomas in Mice Exposed to X-rays and Given Sex Hormones. *Cancer Res.*, **14**, 205.

Gardner, W. U., Dougherty, T. F. & Williams, W. L. (1944) Lymphoid Tumours in Mice Receiving Steroid Hormones. *Cancer Res.*, **4**, 73.

Kaplan, H. S. (1948) Comparative Susceptibility of the Lymphoid Tissues of Strain C57 Black Mice to the Induction of Lymphoid Tumors by Irradiation. *J. natn. Cancer Inst.*, **8**, 191.

Karande, K. A., Mistry, P. B. & Rangan, S. R. S. (1968) Chemical Induction of Mammary Cancer in Pseudopregnant Mice of Strain Strong A. *Ind. J. exp. Biol.*, **6**, 70.

Karande, K. A., Sheth, N. A. & Ranadive, K. J. (1970) Follicle Stimulating Hormone Content of Pituitary Glands of Intact and Ovariectomized Mice of Different Strains. *J. Endocr.*, **48**, 457.

Kirschbaum, A. (1953) Synergistic Action of Leukemogenic Agents. *Cancer Res.*, **13**, 263.

Kirschbaum, A., Shapiro, J. R. & Mixer, H. W. (1949) Synergistic Action of Estrogenic Hormone and X-rays in Inducing Thymic Lymphosarcoma of Mice. *Proc. Soc. exp. Biol. Med.*, **72**, 632.

Kirschbaum, A., Strong L. C. & Gardner, W. U. (1940) Influence of Methylcholanthrene on Age Incidence of Leukaemia in Several Strains of Mice. *Proc. Soc. exp. Biol. Med.*, **45**, 287.

Liebelt, A. G. & Liebelt, R. A. (1962) Influence of Gonadal Hormones and Cortisone on Spontaneous and Methylcholanthrene Induced Leukemia in Inbred Mice. *Cancer Res.*, **22**, 1180.

Meites, J. & Nicoll, C. S. (1956) Adenohypophysis: Prolactin. In V. E. Hall, A. C. Giese and R. R. Sonnenschein (Eds.). *A. Rev. Physiol.*, **28**, 57.

Muhlbock, O. & Boot, L. M. (1959) Induction of Mammary Cancer in Mice without the Mammary Tumour Agent by Isografts of Hypophyses. *Cancer Res.*, **19**, 402.

MURPHY, E. D. (1963) SJL/J, a New Inbred Strain of Mouse with a High, Early Incidence of Reticulum Cell Sarcoma. (Abstract). *Proc. Am. Ass. Cancer Res.*, **4**, 46.

PAI, S. R. & RANADIVE, K. J. (1971) Biological Studies on Leukaemia in the ICRC Mouse: I. Effect of Gonadectomy. *Ind. J. Cancer*, **8**, 92.

PAI, S. R. & RANADIVE, K. J. (1973) Histopathology of Spontaneous Neoplasms of Reticular Tissues in Inbred Line of Albino " ICRC Mouse ". *Ind. J. Cancer*, **10**, 172.

RANADIVE, K. J. & HAKIM, S. A. (1958) *The Chemical Induction of Mammary Cancer in Different Inbred Strains of Mice.* Proc. 2nd Int. Symp. on Mammary Cancer, Perguia, Italy. Ed. L. Severi, p. 441.

RANADIVE, K. J. & KANEKAR, S. A. (1963) Biological Studies on the New Albino Mouse Inbred at the Indian Cancer Research Centre. *Ind. J. med. Res.*, **51**, 1005.

RANADIVE, K. J. & KARANDE, K. A. (1963) Studies on 1 : 2 : 5 : 6 Dibenzanthracene Induced Mammary Carcinogenesis in Mice. *Br. J. Cancer*, **17**, 272.

RANADIVE, K. J. & KARANDE, K. A. (1970) Pituitary of Spayed Mice of Different Strains: Cytology and Gonadotrophic Content. *J. Endocr.*, **48**, 449.

RANADIVE, K. J. & WARAVDEKAR, S. S. (1955) The Influence of " Milk Borne Tumour Agent " on some Endocrine Glands in Castrate Mice. *Proc. Ind. Acad. Sci.*, **52**, 162.

RANADIVE, K. J., KARANDE, K. A. & D'COSTA, V. (1972) Spontaneous Lesions in the New Inbred " ICRC " mouse and its $F_1$ Cross with C3H(Jax). *Ind. J. Cancer*, **9**, 14.

RAPPAPORT, H. & BARONI, C. (1962) A Study of the Pathogenesis of Malignant Lymphoma Induced in the Swiss Mouse by 7:12-dimethylbenzanthracene Injected at Birth. *Cancer Res.*, **22**, 1067.

RASK-NIELSEN, R. (1948) On the Development of Tumours in Various Tissues in Mice Following Direct Application of a Carcinogenic Hydrocarbon. *Acta path. microbiol. scand.*, Suppl. 78.

RASK-NIELSEN, R. (1949) Investigations into the Varying Manifestations of Leukaemic Lesions following Injections of 9:10-dimethyl-1:2-benzanthracene into Different Subcutaneous Sites in Street Mice. *Br. J. Cancer*. **3**, 549.

RASK-NIELSEN, R. (1950) On the Susceptibility of the Thymus, Lung, Subcutaneous and Mammary Tissues in Strain Street Mice to Direct Application of Small Doses of Four Different Carcinogenic Hydrocarbons. *Br. J. Cancer*, **4**, 108.

RUDALI, G., JULIARD, L. & DESORMEAUX, B. (1956) Action cancérigène du méthylcholanthrène et de l'oestradiol sur les souris NLC mâles. *C.R. Soc. Biol.*, **150**, 1853.

SHISHA, H. & NISHIZUKA, Y. (1971) Determining Role of Age and Thymus in Pathology of 7:12-dimethylbenzanthracene induced Leukaemia in Mice. *Gann*, **62**, 407.

SILBERBERG, M. & SILBERBERG, R. (1949) Malignant Lymphoid Tumours in Orchidectomized Mice receiving Hypophyseal and Ovarian Grafts at Various Ages. *Proc. Soc. exp. Biol. Med.*, **72**, 547.

STICH, H. F. (1960) Chromosomes of Tumour Cells: I. Murine Leukaemias Induced by One or Two Injections of 7:12-dimethylbenzanthracene. *J. natn. Cancer Inst.*, **23**, 649.

TOTH, B., RAPPAPORT, H. & SHUBIK, P. (1962) Accelerated Development of Malignant Lymphomas in AKR Mice Injected at Birth with 7:12-dimethylbenz(a)anthracene. *Proc. Soc. exp. Biol. Med.*, **110**, 881.

TOTH, B., RAPPAPORT, H. & SHUBIK, P. (1963) Influence of Dose and Age on the Induction of Malignant Lymphomas and other Tumours by 7:12-dimethylbenz(a)anthracene in Swiss Mice, *J. natn. Cancer Inst.*, **30**, 723.

YUMOTO, T. & DMOCHOWSKI, L. (1967) Light and Electron Microscope Studies of Organs and Tissue of SJL/J Strain Mice with Reticulum Cell Neoplasms Resembling Hodgkin's Disease. *Cancer Res.*, **27**, 2098.